# The *Escherichia coli* COG1738 Member YhhQ Is Involved in 7-Cyanodeazaguanine (preQ_0_) Transport

**DOI:** 10.3390/biom7010012

**Published:** 2017-02-08

**Authors:** Rémi Zallot, Yifeng Yuan, Valérie de Crécy-Lagard

**Affiliations:** Department of Microbiology and Cell Science, Institute of Food and Agricultural Sciences, University of Florida, Gainesville, FL 32611, USA; yuanyifeng@ufl.edu

**Keywords:** 7-deazapurine, queuosine, preQ_0_, preQ_1_, queuine, tRNA modification, transport, salvage, COG1738, ECF-type ATP-binding cassette

## Abstract

Queuosine (Q) is a complex modification of the wobble base in tRNAs with GUN anticodons. The full Q biosynthesis pathway has been elucidated in *Escherichia coli*. FolE, QueD, QueE and QueC are involved in the conversion of guanosine triphosphate (GTP) to 7-cyano-7-deazaguanine (preQ_0_), an intermediate of increasing interest for its central role in tRNA and DNA modification and secondary metabolism. QueF then reduces preQ_0_ to 7-aminomethyl-7-deazaguanine (preQ_1_). PreQ_1_ is inserted into tRNAs by tRNA guanine_(34)_ transglycosylase (TGT). The inserted base preQ_1_ is finally matured to Q by two additional steps involving QueA and QueG or QueH. Most Eubacteria harbor the full set of Q synthesis genes and are predicted to synthesize Q de novo. However, some bacteria only encode enzymes involved in the second half of the pathway downstream of preQ_0_ synthesis, including the signature enzyme TGT. Different patterns of distribution of the *queF*, *tgt*, *queA* and *queG* or *queH* genes are observed, suggesting preQ_0_, preQ_1_ or even the queuine base being salvaged in specific organisms. Such salvage pathways require the existence of specific 7-deazapurine transporters that have yet to be identified. The COG1738 family was identified as a candidate for a missing preQ_0_/preQ_1_ transporter in prokaryotes, by comparative genomics analyses. The existence of Q precursor salvage was confirmed for the first time in bacteria, in vivo, through an indirect assay. The involvement of the COG1738 in salvage of a Q precursor was experimentally validated in *Escherichia coli*, where it was shown that the COG1738 family member YhhQ is essential for preQ_0_ transport.

## 1. Introduction

Queuosine (Q) is a tRNA modification, located at the wobble position of tRNAs Asp, Asn, His and Tyr, found in Eubacteria and Eukaryotes [1]. Modifications of the anticodon loop, contribute to the fidelity and efficiency of protein synthesis [2]. There is evidence that Queuosine does have a role in both of these processes [3,4,5]. Whereas the physiological role of Q is still not fully elucidated, there has been a renewed interest in this molecule, particularly as it appears to be a forgotten micronutrient in humans, discussed in recent reviews [1,6,7,8].

**Figure 1:**
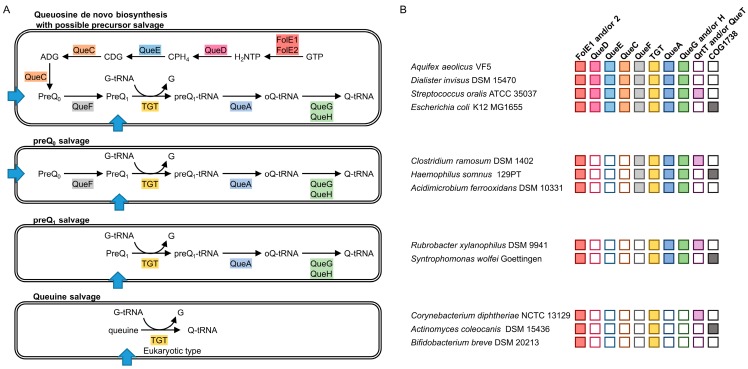
Prediction of Queuosine biosynthesis and salvage pathways. (**A**) Variation among the biosynthesis and salvage capabilities, with predicted transporters represented by blue arrows; (**B**) illustration of the presence of the corresponding genes in representative bacteria. Boxes are colored when the gene is present and empty boxes are for when the gene is absent. Abbreviations: FolE1 and FolE2: GTP cyclohydrolase I (EC 3.5.4.16); QueD*:* 6-carboxytetrahydropterin synthase (EC 4.1.2.50); QueC: 7-cyano-7-deazaguanine synthase (EC 6.3.4.20); QueE: 7-carboxy-7-deazaguanine synthase (EC 4.3.99.3); QueF: NADPH-dependent 7-cyano-7-deazaguanine reductase (EC 1.7.1.13); TGT: tRNA guanine_(34)_ transglycosylase; QueA: the tRNA preQ_1(34)_ S-adenosylmethionine ribosyltransferase-isomerase (EC 2.4.99.17); QueG and QueH: tRNA epoxyqueuosine_(34)_ reductase (EC 1.17.99.6); GTP: Guanosine triphosphate; H_2_NTP: 7,8-dihydroneopterin-3′-triphosphate; CPH_4_: 6-pyruvoyl-5,6,7,8-tetrahydropterin ; CDG: 7-carboxy-7-deazaguanine ; ADG: 7-amido-7-deazaguanine; preQ_0_: 7-cyano-7-deazaguanine; preQ_1_: 7-aminomethyl-7-deazaguanine; preQ_1_-tRNA: preQ_1_ at the position 34 of tRNA; oQ-tRNA: Epoxyqueuosine at the position 34 of tRNA; Q-tRNA: Queuosine at the position 34 of tRNA; G-tRNA: Guanine at the position 34 of tRNA; G: Guanine; NADPH: nicotinamide adenine dinucleotide phosphate; tRNA: transfer RNA.

Queuosine is found in Eubacteria and Eukaryotes, but only Eubacteria can synthesize it [9]. The complete Q biosynthesis pathway has been elucidated in *Escherichia coli* and is conserved in many bacteria (Figure 1A). The guanosine triphosphate (GTP) precursor is converted to 7,8-dihydroneopterin-3′-triphosphate (H_2_NTP) by GTP cyclohydrolase I (EC 3.5.4.16) encoded by the *folE1* or *folE2* genes [10,11]. The first dedicated enzyme of the pathway, 6-carboxytetrahydropterin synthase (EC 4.1.2.50, QueD) converts H_2_NTP to 6-pyruvoyl-5,6,7,8-tetrahydropterin (CPH_4_) [12], before 7-carboxy-7-deazaguanine synthase (EC 4.3.99.3, QueE) catalyzes the formation of 7-carboxy-7-deazaguanine (CDG) [13]. 7-cyano-7-deazaguanine synthase (EC 6.3.4.20, QueC) then produces 7-cyano-7-deazaguanine (preQ_0_) [13] through the recently discovered intermediate 7-amido-7-deazaguanine (ADG) [14]. PreQ_0_ is reduced to 7-aminomethyl-7-deazaguanine (preQ_1_) by the NADPH-dependent 7-cyano-7-deazaguanine reductase (EC 1.7.1.13, QueF) [13]. PreQ_1_ is then exchanged with guanine at the position 34 in target tRNAs by the enzyme tRNA guanine_(34)_ transglycosylase (TGT) [15]. An epoxycyclopentane moiety originating from S-adenosylmethionine is then transferred to the 7-aminomethyl group of preQ_1_ by the tRNA preQ_1(34)_ *S*-adenosylmethionine ribosyltransferase-isomerase (EC 2.4.99.17, QueA) [16]. tRNA epoxyqueuosine_(34)_ reductase (EC 1.17.99.6, QueG) is responsible for the final step, the conversion of epoxyqueuosine (oQ) to Q [17]. It was recently shown that QueG can be replaced by a non-orthologous family, QueH, in specific bacteria [18].

The preQ_0_ intermediate is not only used for Q synthesis. It is also a precursor of the Archaeosine base found in archaeal tRNAs [19,20], of the 7-deazapurine derivatives recently identified in DNA [21] and of secondary metabolites such as toyocamycin and sangivamycin produced by Streptomycetes [22] (for more details, see recent review [8]).

Not all bacteria are capable of Q de novo synthesis [23,24]. Typically, organisms that lack *tgt* homologs also lack the genes involved in the conversion of H_2_NTP to preQ_1_, as well as the genes involved in the maturation of preQ_1_ in tRNAs to Q. These organisms are predicted to never harbor Q in their tRNAs, as seen in *Mycoplasma capricolum* [25].

Other bacteria lack the preQ_0_ or preQ_1_ synthesis genes (*queD*, *queE* and *queC*) but harbor orthologs of the Q signature enzyme TGT, and of various accompanying enzymes, such as QueA and QueG or QueH, and sometimes, QueF. (Figure 1A). These organisms must rely on salvage and the presence of Q in their tRNAs will be dependent on the presence of the precursor bases in the environment [23,24].

Membranes are impermeable to purines; therefore, transporters are required for the salvage of Q precursors. To our knowledge, there is no reported experimental evidence for Q precursor salvage in prokaryotes. However, several strong transporter candidates have been predicted in silico [26,27]. The *qrtT* and *queT* genes encoding substrate-specific integral membrane proteins of ECF-type transporters (Energy-Coupling Factor transporters) are often associated with Q related genes [26,27] and are sometimes located downstream of preQ_1_-responsive riboswitches [28,29]. This strongly suggests a role in preQ_1_ salvage.

In this study, we predicted various patterns for salvage of preQ_0_, preQ_1_ or even queuine from the presence/absence patterns of the Q pathway genes (Figure 1B). We experimentally demonstrated, with an indirect method, the existence of Q precursor salvage in bacteria. In addition, we predicted and experimentally validated that the Clusters of Orthologous Groups 1738 (COG1738) family is a preQ_0_ transporter.

## 2. Results

### 2.1. Analysis of Q Precursors Salvage Capability in Bacteria

tRNA guanine_(34)_ transglycosylase is the signature enzyme of the Q pathway. It catalyzes the critical step that inserts the modified base precursor in tRNAs [15]. It does not have a role in any other pathway, and, to date, it has never been found in organisms that lack Q. However, not all bacteria that harbor TGT encoding genes are able to synthesize Q de novo [23,24]. Figure 1 summarizes the various configurations of Q biosynthesis and salvage pathways that can be predicted in sequenced bacteria.

Several organisms lack the capacity to synthesize the preQ_0_ precursor because the *queD*, *queE* and *queC* genes are missing, even if the genes responsible for the final steps of the pathway, (*queF*, *tgt*, *queA* and *queG* or *queH*) are present and thus, must rely on preQ_0_ or preQ_1_ salvage. Another variation of the above configuration is found where the QueF enzyme, responsible for the conversion of preQ_0_ to preQ_1_, is also absent, making preQ_1_ the only Q precursor that can be salvaged.

A more complex and unexpected variation of Q precursor salvage capability is observed in specific bacteria that only harbor the *tgt* gene. This implies that, similarly to eukaryotes [30], the queuine base is salvaged. If this is the case, the TGT enzymes of these bacteria must have switched their substrate specificity from preQ_1_, classically observed for bacterial enzymes, to queuine, observed in eukaryotic enzymes [31,32]. Sequence alignments of the amino acid sequences of TGT from bacteria harboring this specific salvage configuration do reveal the presence of specific residues that could be responsible for this alternative substrate specificity (Supplementary Figure S1).

### 2.2. Comparative Genomics Identify COG1738 as a Possible Q Precursor Transporter

Queuosine precursors require transporters for import from the external environment for utilization by intracellular salvage enzymes. It is not known if the high affinity transporters for adenine and hypoxanthine/guanine can import Q precursors with low specificity [33]. The substrate-specific integral membrane protein unit of shared ECF transporters genes *qrtT* and *queT* have been predicted to encode for preQ_1_ transporters [26,27], as they are often found physically associated with Q related genes and are sometimes under the control of preQ_1_ riboswitches [28,29]. These genes are clearly found in bacteria that rely on the salvage of Q precursors for the modification to be present in their tRNAs (Figure 1B). However, their transport activity has not been experimentally verified. In addition, not all bacteria relying on Q salvage harbor *qrtT* or *queT* homologs (Figure 1B), implying the existence of other specific transporters for Q precursors.

**Figure 2:**
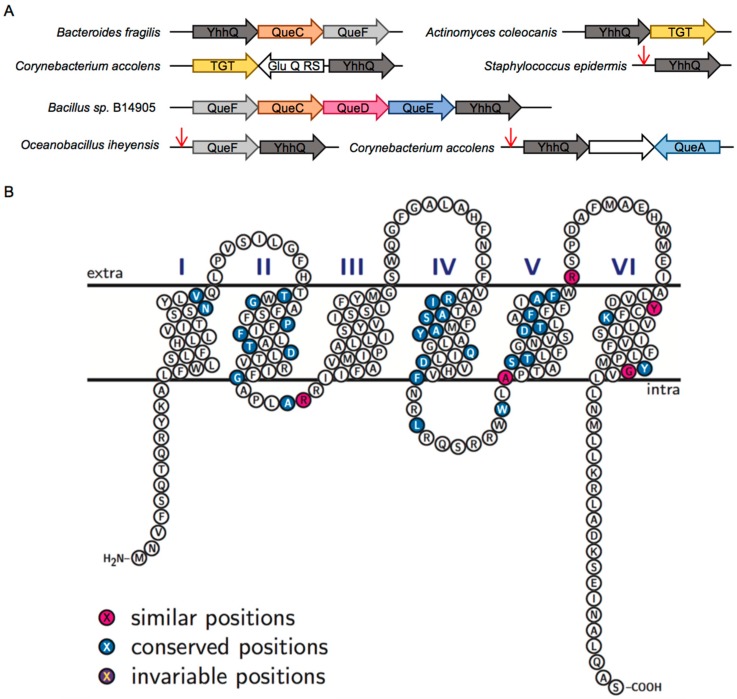
Bioinformatic analysis of YhhQ. (**A**) YhhQ is often found encoded on the genome close to Queuosine related genes. Red arrows represent preQ_1_-responsive riboswitches. GluQ-RS is a Glutamyl queuosine-tRNA synthetase that produces a hyper modification of Queuosine [40]; (**B**) schematic representation of *Escherichia coli* YhhQ (P37619) illustrating the presence of the six proposed transmembrane domains, with standard identity and similarity shading, based on a sequence alignment of sequences defining the COG1738 group (Figure S2 for alignment). Image produced with TexTopo, with information from the P37619 Uniprot entry. Conserved position: ≥50% conservation; invariable: 100% conservation.

Because genes of a given pathway tend to physically cluster in bacterial genomes [34], we investigated the neighborhoods of Q synthesis genes, using the SEED database and its tools [35]. Genes belonging to the COG1738 family, also sometimes abbreviated *yhhQ*, are often found associated with Q related genes (Figure 2A). In addition, *yhhQ* is under the control of various classes of preQ_1_ riboswitches in different bacteria (Figure 2A), as previously reported [28,36], and as shown in the RegPrecise database (under the name YpdP) [37]. Both *yhhQ* and *queE* (*ygcF*) are upregulated by copper in *Erwinia amylovora* [38], reinforcing the link between YhhQ and the queuosine pathway. *YhhQ* is also reported to be a member of the purine regulon (PurR) [39].

The COG1738 family is annotated as an ‘Uncharacterized PurR-regulated membrane protein YhhQ, DUF165 family [Function unknown]’ in the COG database [41] and as a ‘Putative vitamin uptake transporter’ in the protein families database (PFAM) as PF02592 [42]. In the RegPrecise database, YpdP is annotated as a ‘Substrate-specific component YpdP (COG1738) of predicted queuosine-regulated ECF transporter’ [37]. The curated entry in Universal Protein Resource (UNIPROT) for *E. coli* YhhQ (P37619) proposes an inner membrane protein with six transmembrane segments (Figure 2B), characteristic of transporters. The location of this protein embedded in the inner membrane, with its C-terminal tail facing the cytoplasm, has been experimentally validated [43].

The Transporter Classification Database (TCDB) [44] classifies COG1738 among the Vitamin Uptake Transporter (VUT) family (TC# 2.A.88), also presented as an Energy-Coupling Factor (ECF) family. This family encompasses integral membrane proteins that are porters, postulated to capture specific substrates, for which there is minimal evidence for association with an ATP-binding cassette (ABC-type) ATP-hydrolyzing subunit. Indeed, we did not observe any physical clustering association between *yhhQ* genes and genes encoding components from ECF-type ATP-binding cassette transporters. A known example of a vitamin ECF transporter that does not require central ECF components is BioY, which forms homodimers to transport biotin [45]. As ECF central components do not occur in *E. coli* K12 [45] and many other bacteria that harbor YhhQ, it is likely that this is also the case for members of this family.

An alignment of COG1738 proteins from phylogenetically diverse organisms, used as a reference for the definition of the COG1738 group, shows little conservation of specific amino acids (Figure 2B and Figure S2). We propose that subfamilies, possibly with various substrate specificity determinants could exist, and are masking the key residues involved in substrate recognition (see the Sequence Similarity Networks or SSNs below).

Taken altogether, we hypothesize that members of the COG1738 family are transmembrane proteins, with characteristics of transporters, involved in the import of Q precursors.

### 2.3. YhhQ Is Involved in Q Precursor Transport in *E. coli*

*E. coli* is among the organisms that have a complete Q de novo pathway. Even if there is no QtrT or QueT protein encoded by its genome, we predict Q precursors can be salvaged, because of the presence of a YhhQ encoding gene (Figure 1). The rationale for the presence of a salvage pathway in an organism capable of full de novo synthesis is that salvage is more economical than de novo synthesis, if compounds to be salvaged are available in the environment. To test whether *E. coli* can salvage Q precursors, a ∆*queD* strain deficient in preQ_0_ synthesis was used. The role of *E. coli* YhhQ in preQ_0_ and preQ_1_ transport was tested indirectly by following the formation of the Q modification in tRNA^Asp^_GUC_.

**Figure 3:**
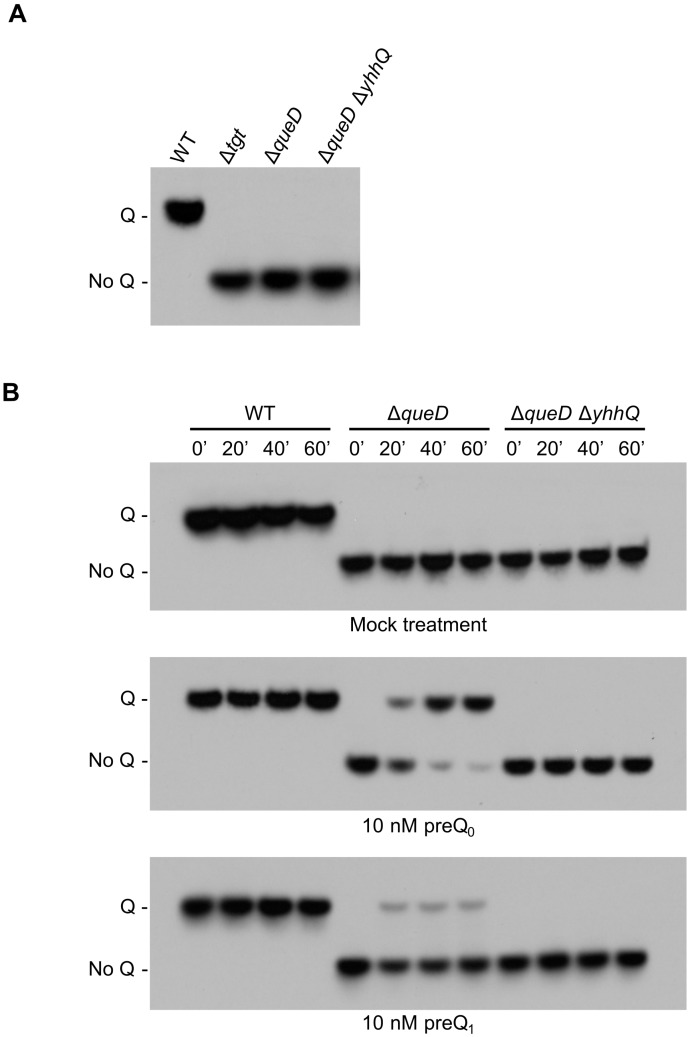
Detection of Q in tRNA^Asp^_GUC_ as a representative of the salvage of the Q precursors preQ_0_ and preQ_1_. *E. coli* bulk tRNAs were separated in an 8 M urea, 8% polyacrylamide gel containing 0.5% 3-(acrylamido)phenylboronic acid and then transferred to a nylon membrane. The transferred tRNAs were probed with a biotinylated primer, and visualized by chemiluminescence. (**A**) tRNAs modified with Q migrate slower than unmodified tRNA, as illustrated with tRNA from Wild Type (WT), and Δ*tgt* grown in Luria-Bertani (LB - positive and negative control, respectively). tRNAs from Δ*queD* and Δ*queD* Δ*yhhQ* grown in defined minimal medium M9 + 0.5% glycerol do not have Q; (**B**) test of the salvage capability of the WT (positive control for Q detection), Δ*queD* and Δ*queD* Δ*yhhQ* strains towards mock (negative control), 10 nM preQ_0_ and 10 nM preQ_1_ treatments. Representative Northern blots shown.

tRNAs modified with Q migrate more slowly in an 8 M urea, 8% polyacrylamide gel containing 0.5% 3-(acrylamido)phenylboronic acid compared to the unmodified tRNA [46]. Following transfer on a nylon membrane, a biotinylated probe is used for the detection of the target tRNA (tRNA^Asp^_GUC_) by Northern blot [4,21]. tRNAs extracted from Wild Type (WT) and Δ*tgt* *E. coli* grown in Luria-Bertani (LB) media were used as positive and negative controls for the presence and absence of Q, respectively (Figure 3A). Preliminary experiments showed that different batches of commercial LB mixes used to make LB broth could be a source of an unknown Q precursor for the Δ*queD* strain thus requiring the need to conduct the salvage experiments in defined minimal M9 medium, supplemented with 0.5% glycerol as carbon source. We verified that tRNAs extracted from the Δ*queD* and Δ*queD* Δ*yhhQ* strains grown in these conditions indeed lacked Q, comparable to that of tRNAs from the Δ*tgt* strain grown in LB broth (Figure 3A).

The presence of Q in tRNA^Asp^_GUC_ was then measured in *E. coli* WT, Δ*queD* and Δ*queD* Δ*yhhQ* strains, after feeding with a mock treatment (negative control) or with 10 nM preQ_0_ or preQ_1_ when cells had reached an optical density (A_600nm_) of 0.6. The transport reaction was stopped at time points of 0, 20, 40 and 60 min by placing samples on melting ice, and then centrifuging, followed by immediate resuspension of cell pellets in Trizol for tRNA extraction. As expected, Q was detected in the WT strain, and was absent from the de novo biosynthesis deficient strains Δ*queD* and Δ*queD* Δ*yhhQ* when no precursors are added. However, when Δ*queD* and Δ*queD* Δ*yhhQ* cells were fed with 10 nM preQ_0_ or preQ_1_, Q is formed in *yhhQ*^+^ tRNA only, but not in the *yhhQ*^−^ (Figure 3B). Therefore, the Q precursors preQ_0_ and preQ_1_ can be salvaged in *E. coli*. In addition, YhhQ is necessary for the salvage of both precursors and most certainly is responsible for the import step, based on the bioinformatic evidence presented above. In addition, these experiments showed that when given a fixed concentration (10 nM) of precursors, preQ_0_ is preferentially incorporated into Q compared to preQ_1_, suggesting that *E. coli* YhhQ is more specific towards preQ_0_ than preQ_1_.

**Figure 4:**
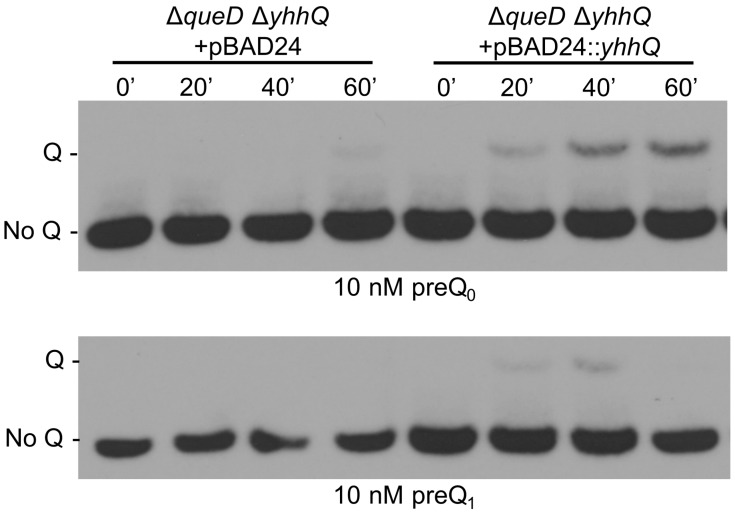
The role of YhhQ as involved in Q precursors salvage is validated by complementation of the Δ*queD* Δ*yhhQ*. Test of the salvage capability of the Δ*queD* Δ*yhhQ* grown in M9 + 0.5% glycerol strains carrying the empty plasmid pBAD24 or pBAD24::*yhhQ* induced with 0.2% arabinose. Representative Northern blots shown.

The complementation of the preQ_0_ transport deficiency of the strain carrying the ∆*yhhQ* allele was tested by transforming the Δ*queD* Δ*yhhQ* strain with a pBAD24::*yhhQ* derivative or the control empty pBAD24 plasmid. The presence of the *yhhQ in trans* restored the salvage of Q in tRNA in the Δ*queD* Δ*yhhQ* background (Figure 4).

In the various conditions tested for the complementation experiments, it was observed that small amounts of Q modified tRNAs can be detected, even in yhhQ^−^ strains, after extended incubation times (Figure S3). This suggests the existence of non-specific transporters for preQ_0_ in *E. coli*. It is possible that the previously characterized purine transporters [33] are able to import deazaguanine derivatives in addition to their canonical substrates.

Taken together, these genetic experiments validate our hypothesis that members of the COG1738 family are involved in the transport of preQ_0_/preQ_1_ for Q salvage. Because the experimental set-up used is only an indirect proof of transport, it is not clear whether unknown partners of YhhQ are involved.

### 2.4. The COG1738 Family Is Not Homogeneous and May Be Involved in the Transport of Other Purines

Even if the *E. coli* YhhQ seems to be more efficient towards PreQ_0_ than preQ_1_, this might not always be the case for other members of the COG1738 family. Indeed, YhhQ homologs are found in organisms that cannot use preQ_0_ because their genomes do not harbor *queF* genes (Figure 1). Moreover, the lack of a universally conserved residue among the whole COG1738 family suggests that this family may be constituted of various functionally related subfamilies (Figure 2B and Figure S2).

**Figure 5:**
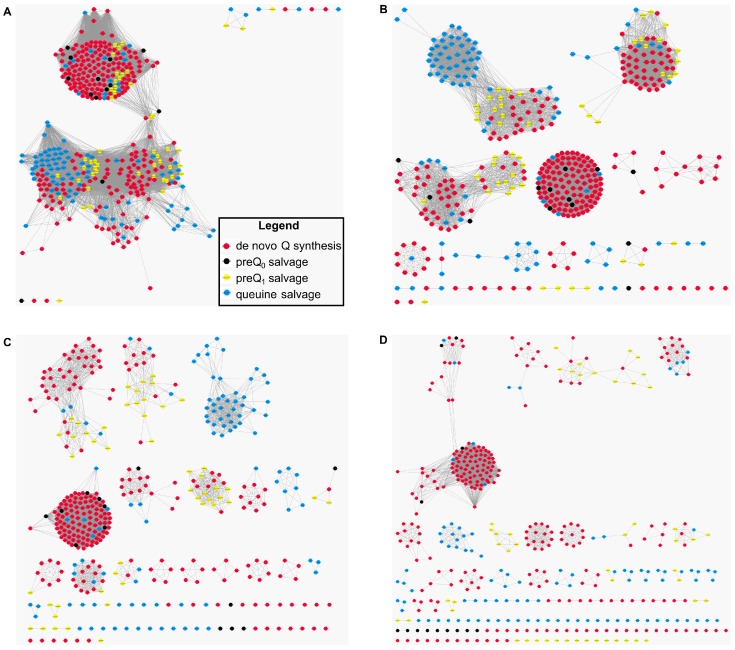
Protein sequence similarity networks (SSNs) for the analysis of the sequence relationships in the COG1738 protein family. YhhQ sequences were obtained from the pubSEED [35] subsystem “Queuosine bacterial salvage”, and colored depending on the configurations of salvage for preQ_0_, preQ_1_, queuine or the queuosine de novo synthesis capability of the organism from which they originate. SSN from a score of 20 (**A**); 40 (**B**); 60 (**C**); or 80 (**D**) tends to show that YhhQ proteins are separated into subfamilies corresponding to the salvage or de novo capability (with a few exceptions).

The variability among YhhQ sequences was explored using the Enzyme Function Initiative-Enzyme (EFI) Similarity Tool [47]. YhhQ sequences were obtained from the manually curated pubSEED [35] subsystem “Queuosine bacterial salvage” and annotated in 1600 diverse organisms according to the various configurations of the Q pathway: preQ_1_, preQ_0_ or queuine salvage or de novo synthesis (Figure 1). Parameters for SSN alignment scores were explored from 20 to 80, by 20 increments, and colored according to the Q salvage configuration from the corresponding organism. With low scores, most of the YhhQ sequences group together, while increasing stringency separates them into subgroups that cluster according to the salvage pathway configuration from the organisms they originate from (Figure 5) (with a few exceptions).

The SSN analysis suggests the existence of specificity determinants among the YhhQ family for preferred salvage of preQ_0_, preQ_1_ or queuine.

## 3. Discussion

A comparative genomics approach revealed the strong association of members of the COG1738 protein family with Q synthesis genes. COG1738 proteins have transmembrane segments typical of transporters. We hypothesized that this protein family was involved in the salvage of the Q precursors preQ_0_, preQ_1_ or queuine. The salvage capacity of preQ_0_ and preQ_1_ by *E. coli* was experimentally demonstrated for the first time through an indirect assay. The prediction that *yhhQ* is involved was also validated. Whether other proteins are required for this transport activity is still to be determined. There is variability among the COG1738 sequences, as clearly seen with an SSN approach, showing the appearance of clearly separated subgroups. We expect the existence of determinants for the specialization of transport towards preQ_0_, preQ_1_ or queuine exist, but further work is required for their identification. As seen here, the Q detection from very little bulk tRNAs with the 3-(acrylamido)phenylboronic acid gel, revealed by non-radioactive Northern blot with great sensitivity, should facilitate future studies.

In addition, proteins belonging to the COG1738 family are found in bacteria capable of de novo synthesis. This suggests that salvage may be preferred in conditions where the corresponding Q precursors are readily available in the environment. Homologs are also found in Archaea, which are not able to synthesize Q but the preQ_0_ derivative archaeosine instead [48]. A preQ_0_ salvage pathway is likely present in these organisms. Similarly, COG1738 proteins are also found in bacteria that lack the TGT responsible for the insertion of preQ_1_ in tRNA. Interestingly, these organisms have the genes encoding for deazapurine DNA modification that uses preQ_0_ [21].

## 4. Materials and Methods 

### 4.1. Comparative Genomics and Bioinformatics

The BLAST tools [49] and resources at the National Center for Biotechnology Information (NCBI) were used. Multiple sequence alignments were built using Clustal Omega [50] or Multalin [51], and visualized with BOXSHADE or TexShade [52]. Analysis of the phylogenetic distribution and physical clustering was performed in the SEED database [35]. The topology of *E. coli* YhhQ (P37619) was done with TexTopo [53], based on the information available on UNIPROT [54]. The COG [41], PFAM [42], RegPrecise [37], and TCDB [44] databases were cross-referenced. SSN were generated with the EFI [47], from a FASTA file containing manually curated YhhQ homologs sequences, extracted from the pubSEED [35] subsystem “Queuosine bacterial salvage”, and marked according to the various configurations of salvage, preQ_1_, preQ_0_ or queuine salvage and de novo synthesis, among 1600 diverse organisms.

### 4.2. Strains and Growth Conditions

For standard procedures, *E. coli* strains were grown in Luria–Bertani medium (LB - Thermo Fisher Scientific, Waltham, MA, USA) at 37 °C. Solid media were prepared with addition of 15 g/L agar (Thermo Fisher Scientific, Waltham, MA, USA). Kanamycin (Kan, 50 μg/mL, Sigma-Aldrich, St. Louis, MO, USA) and ampicillin (Amp, 100 μg/mL, Sigma-Aldrich, St. Louis, MO, USA) were added when required. *E. coli* strain Δ*queD* (VDC2043) and the corresponding Wild Type strain MG1655 were obtained from a previous study [55]. A *yhhQ*::Km^R^ deletion was introduced in the WT strain following standard procedures [56], with primers 5′-TCGTACGTATTGGGTTCCCTCACCCCAATGGTTAATCAAAAAGGTACAATATTCCGGGGATCCGTCGACC-3′ and 5′-CCTTTCGATAAACGGCCATAACGGCTCATTCATCCATCTTATCACAACTCTGATAACGAACCTTGTAGGCTGGAGCTGCTTCG-3′. P1 transduction [57] was then used to move the *yhhQ*::Km^R^ deletion into the Δ*queD* strain, yielding a Δ*queD*Δ*yhhQ::*Km^R^ strain (VDCRGZ4056—elsewhere abbreviated as Δ*queD*Δ*yhhQ*). These strains were verified by PCR. *E. coli* *yhhQ* was cloned into the sites *Nco*I and *Hind*III of pBAD24 using primers 5′-ACATGCCATGGACGTTTTCTCGCAAACT-3′ and 5′-GCCCAAGCTTTTAACTCGCCTGCAAAGCG-3′ following standard procedures.

In preparation for the salvage assays, the WT, Δ*queD* and Δ*queD*Δ*yhhQ* strains were re-streaked three successive times on M9 minimal defined media (Sigma-Aldrich, St. Louis, MO, USA) with 1% glycerol (Thermo Fisher Scientific, Waltham, MA, USA) as a carbon source and solidified with 15 g/L of agarose (Thermo Fisher Scientific, Waltham, MA, USA). In these conditions, Q in tRNA is completely depleted in the Δ*queD* and Δ*queD*Δ*yhhQ* strains, allowing for no background Q detected, and thus suitable for the salvage experiments. The salvage assays were realized in M9 media with 0.5% glycerol as a carbon source, ampicillin for selection, and 0.2% arabinose (Sigma-Aldrich, St. Louis, MO, USA) to induce gene expression in the complementation assays. preQ_0_ or preQ_1_ (from Ark Pharm, Libertyville, IL, USA and Sigma-Aldrich, St. Louis, MO, USA, respectively) were added to cultures when optical density reached 0.6, and this time was considered *t*_0_ for kinetics. For each time point, 2 mL of culture were transferred to a microtube and briefly placed on melting ice. Microtubes were then immediately centrifuged at max speed in a refrigerated (4 °C) bench-top centrifuge for 30 s, and the supernatant was removed.

### 4.3. Bulk tRNA Purification and Q Detection for tRNA^Asp^_GUC_

Bulk tRNA were prepared from cell pellets, resuspended in 1 mL of Trizol (Thermo Fisher Scientific, Waltham, MA, USA). Small RNAs were extracted using Purelink miRNA Isolation kit (Thermo Fisher Scientific, Waltham, MA, USA) according to the manufacturer’s protocol. The purified RNA were eluted in 50 μL of RNase free water. This extraction method was proven efficient for the purification of tRNA enriched fractions [58], elsewhere referenced in this manuscript as tRNAs.

Detection of the presence of Q in tRNA was adapted from a protocol developed by Igloi and Kossel [46] and recently used by Zaborske et al. [4] and Thiaville et al. [21]. For each sample, bulk tRNAs were deacylated by incubation in 100 mM Tris-HCl (pH 9—prepared at room temperature, Thermo Fisher Scientific, Waltham, MA, USA), for 30 min at 37 °C. Deacylated tRNAs were precipitated using ammonium acetate, isopropanol, and linear polyacrylamide as a carrier [59]. The pellet obtained was washed with 70% ethanol and dried in a Vacuum Concentrator System (Labconco, Kansas City, MO, USA) at 40 °C for 10 min. Prepared tRNAs were resuspended in RNase free water and quantified using a Nanodrop 1000 spectrophotometer. For each lane, 120 ng of tRNAs were resuspended in RNA Loading Dye (NEB, Ipswich, MA, USA) and loaded onto a denaturing 8 M urea (Thermo Fisher Scientific, Waltham, MA, USA), 8% polyacrylamide gel (Thermo Fisher Scientific, Waltham, MA, USA) containing 0.5% 3-(Acrylamido)phenylboronic acid (Sigma-Aldrich, St. Louis, MO, USA). The migration was performed at 4 °C in 40 mM Tris, 20 mM acetic acid, and 1 mM EDTA pH 8.3 (1X TAE—Sigma-Aldrich, St. Louis, MO, USA). Migrated tRNAs were transferred onto a Biodyne B precut Nylon membrane (Thermo Fisher Scientific, Waltham, MA, USA) using a wet transfer apparatus in 1X TAE at 150 mA 4 °C for 90 min. After the transfer, the membrane was baked in an oven for 30 min at 80 °C and then ultraviolet (UV) irradiated in a UV Crosslinker (Fisher FB-UVXL-1000, Thermo Fisher Scientific, Waltham, MA, USA) at a preset UV energy dosage of 120 mJ/cm^2^. tRNA^Asp^_GUC_ was detected with the North2South Chemiluminescent Hybridization and Detection Kit (Thermo Fisher Scientific, Waltham, MA, USA). The initial membrane blocking was realized with DIG Easy Hyb (Roche, Basel, Switzerland) because it drastically limits the background noise compared to the membrane blocking buffer supplied with the North2South kit (Thermo Fisher Scientific, Waltham, MA, USA). Hybridization was done at 60 °C, while using the specific biotinylated primer for tRNA^Aap^_GUC_ [60] (5′-biotin-CCCTCGGTGACAGGCAGG-3′) at 0.3 μM final. The blot was exposed to X-Ray film (Thermo Scientific, CL-X Posure Film) for 5 s. The film was developed using a film processor (Konica QX-60A, Tokyo, Japan).

## 5. Conclusions

This study is the first to demonstrate in vivo salvage of Q precursors in bacteria, with YhhQ involved in the transport, and constituting the third transport system identified. However, some bacteria that have to rely on salvage of Q precursors to have Q in their tRNAs do not have genes encoding for YhhQ, QtrT or QueT transporters in their genomes (Figure 1B and ‘Queuosine bacterial salvage’ subsystem). Possibly, other transport systems exist and remain to be discovered.

The COG1738 protein family is detected in a very limited number of eukaryotic genomes (only four). We thus can exclude this family as being a major player in the eukaryotic transport of salvageable forms of queuosine. The identity of the transporter(s) expected to be involved in queuosine salvage in eukaryotes remains elusive.

## Supplementary Materials

The following are available online at www.mdpi.com/2218-273X/7/1/12/s1, Figure S1: Sequence alignment of Sequence alignment of tRNA guanine_(34)_ transglycosylase (TGT) proteins, Figure S2: Sequence alignment of Clusters of Orthologous Groups 1738 (COG1738) proteins, Figure S3: Non-specific transport of 7-cyano-7-deazaguanine (preQ_0_).
