# Supplementary material for: The Escherichia coli COG1738 Member YhhQ Is Involved in 7-Cyanodeazaguanine (preQ0) Transport
Source: Biomolecules. 2017 Feb 8;7(1):12. doi: 10.3390/biom7010012 (PMC5372724; doi:10.3390/biom7010012)
Supplement: Supplementary file 1 [file biomolecules-07-00012-s001.pdf]

Supplementary Materials: The *Escherichia coli* COG1738 Member YhhQ Is Involved in 7-Cyanodeazaguanine (preQ$_0$) Transport

Rémi Zallot, Yifeng Yuan and Valérie de Crécy-Lagard

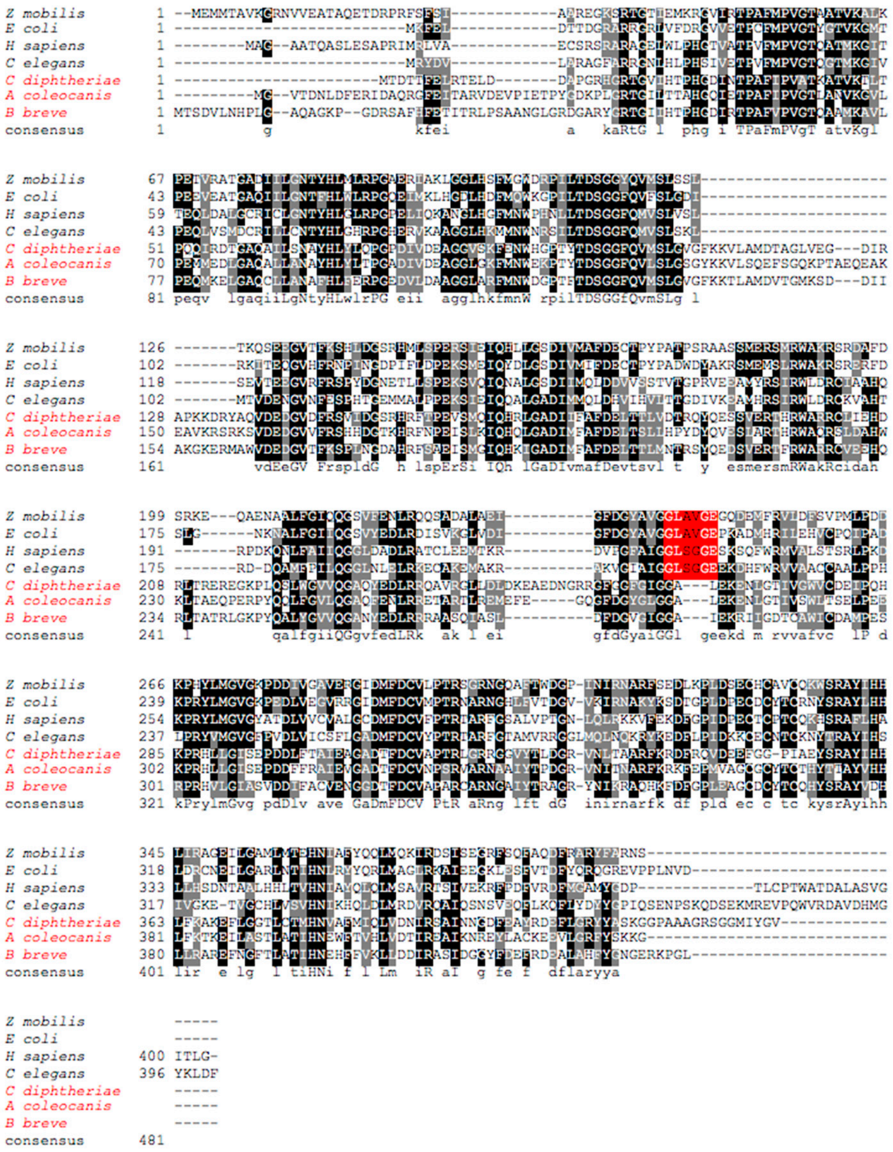

Figure S1. Sequence alignment of tRNA guanine$_{(34)}$ transglycosylase (TGT) proteins. Bacteria that do not have queuosine biosynthesis capability, but have TGT, and not QueA (tRNA preQ$_{1(34)}$ S-adenosylmethionine ribosyltransferase-isomerase (EC 2.4.99.17)), QueG or QueH (tRNA epoxyqueuosine$_{(34)}$ reductase (EC 1.17.99.6)) (with species names in red), have non-canonical substrate binding residues in their TGT sequence. For reference, the substrate binding pockets of the different TGT families that have been well characterized are presented in red boxes [32]. Alignment realized with Clustal Omega [50]. Abbreviations: *Z mobilis*: *Zymomonas mobilis*; *E coli*: *Escherichia coli*; *H sapiens*: *Homo sapiens*; *C elegans*: *Caenorhabditis elegans*; *C diphtheria*: *Corynebacterium diphtheria*; *A coleocanis*: *Actinomyces coleocanis*; *B breve*: *Bifidobacterium breve*.

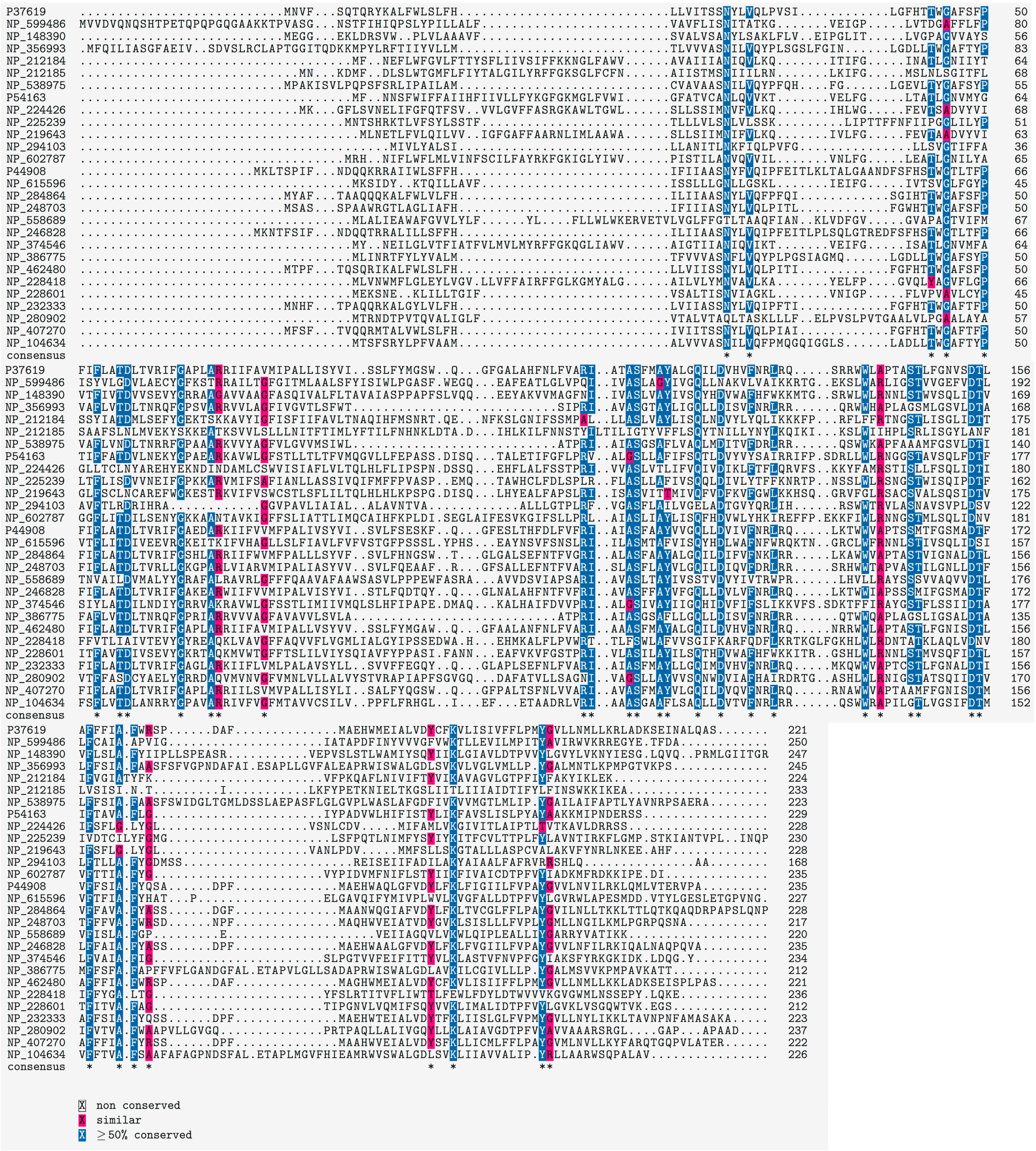

Figure S2. Sequence alignment of COG1738 proteins. Alignment of members of the COG1738 group realized with Clustal Omega [50], and shading with TexShade [52]. P37619: *Escherichia coli* K12; NP 599486: *Corynebacterium glutamicum* ATCC 13032; NP_148390: *Aeropyrum pernix* K1; NP_356993: *Agrobacterium tumefaciens* C58; NP_212184: *Borrelia burgdorferi* B31; NP_212185: *Borrelia burgdorferi* B31; NP_538975: *Brucella melitensis* 1 16M; P54163: *Bacillus subtilis* 168; NP_224426: *Chlamydophila pneumoniae* CWL029; NP_225239: *Chlamydophila pneumoniae* CWL029; NP_219643: *Chlamydia trachomatis* D/UW-3/CX; NP_294103: *Deinococcus radiodurans* R1; NP_602787: *Fusobacterium nucleatum* ATCC 25586; P44908: *Haemophilus influenza*; NP_615596: *Methanosarcina acetivorans* C2A; NP_284864: *Neisseria meningitidis* Z2491; NP_248703, *Pseudomonas aeruginosa* PAO1; NP_558689, *Pyrobaculum aerophilum* IM2; NP_246828: *Pasteurella multocida* Pm70; NP_374546: *Staphylococcus aureus* N315; NP_386775: *Sinorhizobium meliloti* 1021; NP_462480: *Salmonella enterica* LT2; NP_228418: *Thermotoga maritima* MSB8; NP_228601: *Thermotoga maritima* MSB8; NP_232333: *Vibrio cholerae* O1 N16961; NP_280902: *Halobacterium* NRC-1; NP_407270: *Yersinia pestis* CO92; NP_104634: *Mesorhizobium loti* MAFF303099.

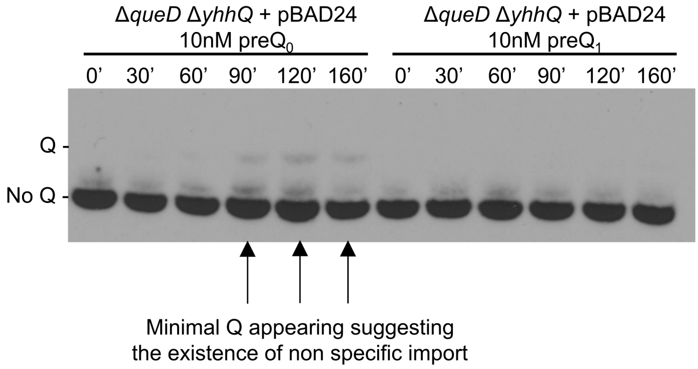

Figure 3. Non-specific transport of 7-cyano-7-deazaguanine ($preQ_0$). Detection of Queuosine in $tRNA^{Asp}_{GUC}$ as a representative of the salvage of the Q precursors $preQ_0$ and 7-aminomethyl-7-deazaguanine ($preQ_1$). *E. coli* bulk tRNA was separated on an 8 M urea, 8% polyacrylamide gel containing 0.5% 3-(acrylamido)phenylboronic acid and transferred to a nylon membrane. The transferred tRNA was probed with a biotinylated primer, and detected by Northern blot. At long incubation times, Q is detected in tRNAs extracted from the *ΔqueD ΔyhhQ* strain (carrying pBAD24), when fed with 10 nM $preQ_0$, suggesting the presence of low-affinity transporters.
